# Edible insects and legumes exert an antioxidant effect on human colon mucosal cells stressed with 2,2′-azobis (2-amidinopropane)-dihydrochloride

**DOI:** 10.3389/fnut.2023.1219837

**Published:** 2023-07-06

**Authors:** Veronica D’Antonio, Natalia Battista, Carla D. Di Mattia, Giampiero Sacchetti, Marina Ramal-Sanchez, Roberta Prete, Donato Angelino, Mauro Serafini

**Affiliations:** Department of Bioscience and Technology for Food, Agriculture and Environment, University of Teramo, Teramo, Italy

**Keywords:** edible insects, bugs, legumes, pulses, oxidative stress, free radicals, reactive oxygen species

## Abstract

**Introduction:**

Edible insects have been recognized as a more sustainable source of nutrients and bio-active compounds than animal-based products, in line with classical vegetable sources such as legumes. In this study, we assessed the antioxidant properties of four edible insects (silkworms, grasshoppers, mealworms and giant worms) and four legume seeds (lentils, chickpeas, *Roveja* peas and grass peas).

**Methods:**

After the aqueous extraction or *in vitro* simulated digestion process, selected products were assessed for: (i) *in vitro* antioxidant capacity through Ferric Reducing Antioxidant Power (FRAP) assay; (ii) the ability to reduce free radicals production induced by a pro-oxidant agent in cells of human colonic mucosa.

**Results:**

All the aqueous extracts and digesta of edible insects displayed significantly higher *in vitro* antioxidant activity than legumes. Moreover, edible insects at all tested concentrations were able to exert an antioxidant effect in the cellular model, while legumes were effective mainly at high concentrations.

**Discussion:**

Despite human trials are need to confirm and define these results in a physiological situation, here we suggest a role for edible insects in oxidative stress prevention. Since oxidative stress is strongly correlated with several intestinal pathologies, the results obtained could be interesting for the prevention and relief of the negative symptoms, offering new advantages to their already known ecological and nutritional properties.

## 1. Introduction

World’s population is expected to reach about 11 billion in 2100 (1), implying an increase in the resources necessary to meet the adequate nutritional requirements of the population. Currently, food production has an already remarkable impact on the environment, accounting for more than the 26% of the global greenhouse gas (GHG) emissions. Indeed, on-farm raising of animals—intended for meat, dairy, eggs, and seafood production—accounts alone for the 31% out of the total food emissions (2). These values are even more alarming if considering that the intake of such animal-based foods is globally higher with respect to the reference dietary intakes (3). Therefore, the interest in alternative foods with a low ecological impact and adequate nutritional and functional properties has considerably increase, to substitute or complement the animal-based products.

The use of insects as food is widely reported in human history (4), and nowadays are currently consumed in zones with the highest number of edible insect species, as China, India, and Mexico, as well as the subtropical and tropical regions of the world (4, 5). From a nutritional point of view, several species are considered as an optimal and potential source of proteins, accounting for approximately the 35% of dry matter (d.m.) of Isoptera and 61% d.m. of Orthoptera (6). Lipids represent the second largest fraction, with a content ranging from 10 to 50% on dry basis, depending on the life stage and the species. Edible insects are also a valuable source of essential fatty acids, as linoleic and α-linolenic fatty acids (7), presenting a higher concentration of linoleic acid compared with salmon (8). Edible insects contain also a significant amount of fiber, different from vegetable sources. In fact, the most abundant fiber is the exoskeletal chitin, which may have a beneficial impact on health by selectively promoting the growth of beneficial bacterial species in the intestine, and thus is currently under investigation (9). More specific information about the nutritional profile of different species of edible insects has been widely described (5, 10).

From an ecological point of view, edible insects have a conceivable role as a sustainable replacement of meat and animal products: their rearing has several advantages with respect to conventional livestock in terms of global warming potential, and land (11) and water (12) used, which are lower when obtaining 1 kg of edible protein from insects in comparison with chicken, pork, or beef.

Besides the undoubted ecological advantages and nutritional properties, it has been demonstrated that edible insects are able to exert also functional effects (13). For instance, their modulation of the redox system has generated a high interest in research, as recently reviewed (14). These properties were recorded *in vitro* and in cellular or animal models; the antioxidant capacity was more evident when specific stressors were present, including dietary ones.

In order to propose the insects as a reasonable alternative to traditional animal products, a comparison with foods presenting a low ecological impact and similar nutritional values is required. In this regard, legumes represent a suitable benchmark thanks to their properties. Their importance—and generally the consumption of plant-based foods—for the health promotion of the planet and individuals are globally established (15); in particular, it is well known that legumes contain bioactive compounds (16, 17) with *in vitro* antioxidant activity (17). Furthermore, consumption of legumes (or their fraction) is able to modulate the oxidative stress *in vivo* (18), representing an optimal alternative to animal products. In this study, we assessed the antioxidant properties *in vitro* of four edible insects, as silkworms (*Bombix mori*), grasshoppers (Orthoptera), mealworms (*Tenebrio molitor*), and giant worms (*Zophobas morio*), and of four legumes, i.e., lentils (*Lens culinaris*), chickpeas (*Cicer aretinum*), *Roveja* peas (*Pisum sativum*), and grass peas (*Latyrus sativus*), using a widely-used human colon mucosal cell line.

## 2. Materials and methods

A graphical representation of the considered insect and legume samples, the extraction and digestion procedures and the relative cell treatments has been provided in Supplementary Figure 1.

### 2.1. Samples

Mealworms (*Tenebrio molitor*—100 g), grasshoppers (Orthoptera—250 g), silkworms (*Bombix mori*—250 g), and giant worms (*Zophobas morio*—100 g), in dried forms, were purchased from Next Food (FZE, Ras Al Khaima, United Arab Emirates). Packs of 500 g of dried *Roveja* peas, chickpeas, lentils, and grass peas were purchased by a local vendor in Teramo (Italy) in September 2020. Seeds and insects were stored in the dark at room temperature.

Prior to extraction or digestion, legumes were soaked overnight in tap water 1:3 (w/v) and then boiled in sink water 1:9 (w/v). The boiling times were 30 min for grass peas, 40 min for lentils, 60 min for *Roveja*, and 120 min for the chickpeas. Boiled seeds were stored in an airtight container at −20°C until their use. Wings and paws of grasshoppers were removed and discharged.

### 2.2. Dry matter and extracts

Dry matter of samples was determined according to the gravimetrical method. Aqueous extracts were performed according to Di Mattia et al. (19), with slight modifications. Briefly, samples were grinded using a Precellys Evolution homogenizer, and the defatting procedure was carried out with 4 g of grounded samples mixed and vortexed with 25 mL of hexane, and then centrifuged at 2,346 × *g* at 4°C. This procedure was repeated three times, discarding the supernatant after each cycle. The lipid-free solids were dried under helium efflux until the complete removal of the hexane. Then, 1 g of the dried lipid-free fraction was added to 25 mL of bi-distilled water, vortexed for 1 min, and shacked for 1 h at 18°C under dark conditions. The homogenate was then centrifuged for 15 min at 2,346 × *g*, and, once the supernatant was filtered, bi-distilled water was added to reach a final volume of 25 mL. The extracts were stored at -20°C until the beginning of the experiment.

### 2.3. Digestion procedures

Digestion protocol referred to the harmonized INFOGEST static *in vitro* digestion procedure, simulating the physiological conditions of the oral, gastric, and small intestinal digestion phases *in vitro* (20), with opportune modifications.

The oral phase was carried out by using human saliva collected from healthy volunteers, according to Chen et al. (21). The fresh saliva samples were collected after 2 h from the last meal. The donors were invited to rinse their mouth with deionized water for at least 30 s to obtain a neutral environment and then saliva from the first 30 s was discarded. Saliva was collected in the next 5 min each 30 s, until the needed amount was reached. The collected saliva was immediately centrifuged at 2,346 × *g* for 10 min and the supernatant stored at −20°C.

In order to simulate mastication, 1 mL of human saliva was added to 1 g of insect or cooked legumes and then the mixture was grinded with mortar and pestle for 2 min. Then, final volume of 2 mL was reached with deionized water. The gastric phase was started by adding simulated gastric fluid containing 2,000 U/mL of pepsin in the final volume. pH was adjusted to 3 and volume to 4 mL prior to incubate the mixture at 37°C for 2 h in a rotating mixer. Then, a solution containing simulated intestinal fluid, containing bile extract (10 mM of bile salts in the final volume) and pancreatin (100 U/mL of trypsin activity in the final volume) was added. The pH was adjusted to 7 and the volume to 8 mL, and the mixture was incubated overnight at 37°C in a rotating mixer. The final products of the subsequent application on each sample of oral, gastric, and intestinal digestion were collected, filtered through cellulose filters of 0.20 μm, aliquoted, and stored at −20°C until cell treatments.

### 2.4. Ferric reducing antioxidant power

The reducing activity of the samples was determined according to the method described by Benzie and Strain (22), with some modifications. Ferric reducing antioxidant power (FRAP) reagent was prepared by mixing acetate buffer (300 mM, pH 3.6), 10 mM 2,4,6-tripyridyl-s-triazine solubilized in 40 mM HCl, and 20 mM FeCl_3_ at 10:1:1 ratio. Samples were thawed and prepared by filtering through 0.20 μm filters. In a 96-well microplate, 20 μL of diluted sample or standard were added to 130 μL of FRAP reagent. A calibration curve based on FeSO_4_·7H_2_O was used. After 30 min of incubation at 37°C, absorbance at 539 nm was recorded by using an EnSpire Multimode Plate Reader (PerkinElmer, Waltham, MA, United States). Results were expressed as mmoles of Fe^2+^ per 100 grams of dry matter (d.m.).

### 2.5. Cellular redox status assessment

Cellular redox status was assessed by dichlorofluorescein diacetate (DCF-DA) assay, as reported by Finamore et al. (23). Normal human colon mucosal epithelium cell line (NCM460) from INCELL Corporation, LLC (San Antonio, TX, United States) were grown in DMEM (Corning, NY, United States) supplemented with 1% (v/v) Penicillin/Streptomycin 100 × (Corning, NY, United States), 1% (v/v) Non-Essential Amino Acids 100 × solution (Corning, NY, United States), and 10% (v/v) heated inactivated Fetal Bovine Serum (Corning, NY, United States). Cell cultures were maintained at 37°C in a humified saturated atmosphere with 5% CO_2_ in culture dishes, and seeded then in 96-well plates for 24 h prior to the experiments. Cells were then washed with 100 μL Hanks’ Balance Salt Solution (HBSS)/well and incubated with 10 μM DCF-DA for 30 min at 37°C, then washed. After that, cells were treated with prooxidant 2,2′-azobis (2-amidinopropane) dihydrochloride (ABAP) in HBSS solution, in combination with water extracts or digested samples at different concentrations. Prior to the beginning of analysis, thawed samples were filtrated through 0.20 μm filters and applied at 0.4, 0.8, 1.6, and 8 g d.m./L, while final concentration of ABAP was 250 μM. In a preliminary experiment, it was assessed that digestion fluids and enzymes present in the digested samples at the applied concentrations did not show any antioxidant capacity in this model (data not shown). For each single experiment, HBSS was used as a blank, whereas cells treated with only HBSS, only ABAP or ABAP in combination with 2 mM ascorbic acid (ABAP + ascorbic acid, AA) were used as controls. 2′,7′ dichlorofluorescein (DCF) fluorescence was monitored every 5 min for 1 h by using an EnSpire Multimode Plate Reader (PerkinElmer, Waltham, MA, United States) at excitation and emission wavelengths of 485 and 535 nm, respectively. Results were expressed as percentage of fluorescence unit over respect to fluorescence unit measured for cells treated only with ABAP.

### 2.6. Data analysis

Data analysis was performed using Prism 8.0.1 program (GraphPad Software Inc., La Jolla, CA, United States). Results were expressed as mean ± SEM of three independent experiments. Differences were considered to be significant at a value of *p* ≤ 0.05, according to one-way ANOVA followed by Bonferroni’s *post hoc* analysis.

## 3. Results

### 3.1. Edible insects showed a higher reducing ability by FRAP compared with legumes

**Figure 1:**
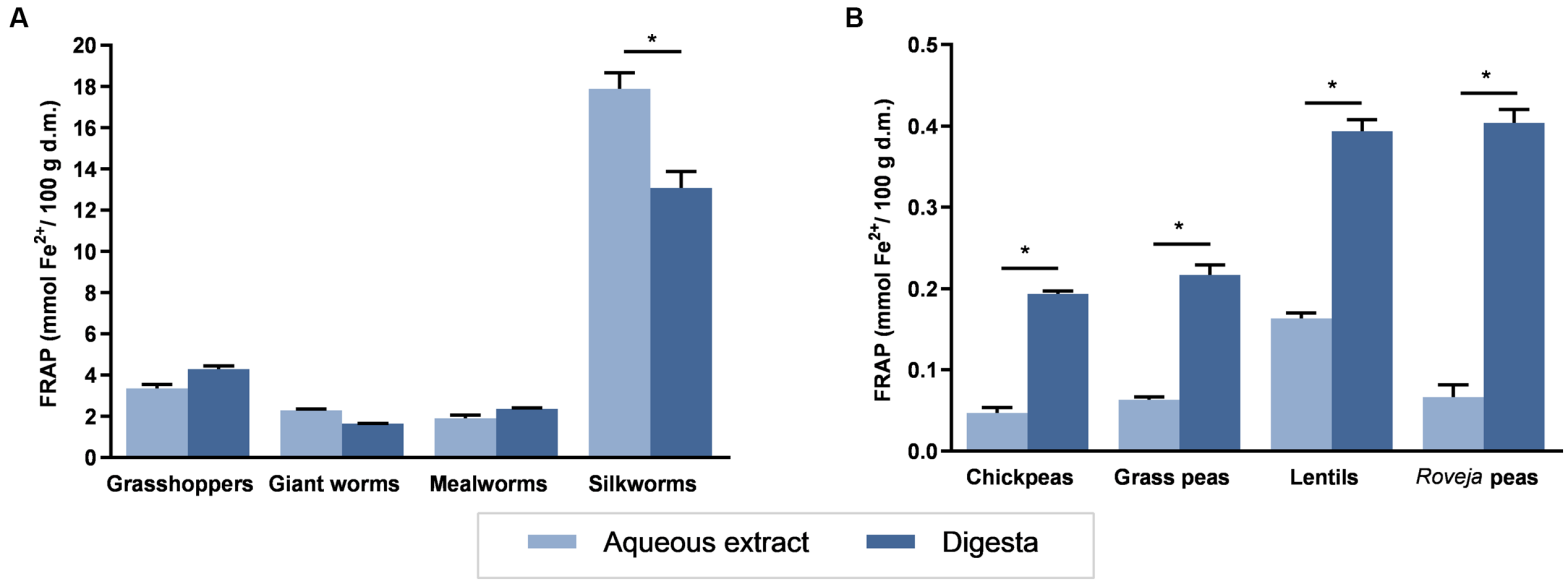
Ferric reducing antioxidant potential (FRAP) of food extracts. FRAP values for aqueous extracts or digesta of edible insects **(A)** or cooked legumes **(B)** are expressed as mmol Fe^2+^/100 g d.m. Each column represents the mean ± SEM (*n* = 3). ^*^p < 0.05 aqueous extract versus corresponding digesta, according to one-way ANOVA followed by Bonferroni’s *post hoc* analysis.

Figure 1 reported the FRAP of aqueous extracts or digesta of edible insects or cooked legumes. Edible insects showed FRAP values ranging from 1.90 (mealworms) to 17.89 (silkworms) mmol Fe^2+/^100 g d.m. for aqueous extracts and 1.65 and 13.0 mmol Fe^2+/^100 g d.m. (giant worms and silkworms, respectively) for digesta. Legumes reported lower ranges: from 0.05 to 0.17 mmol Fe^2+^/100 g d.m. for aqueous extracts and from 0.19 to 0.40 mmol Fe^2+^/100 g d.m. for digesta, with lower values corresponding to chickpeas and the highest to lentils. Aqueous extracts of silkworm showed the highest reducing ability among all the samples, while chickpeas showed the lower FRAP value. The superior reducing activity of edible insects with respect to legumes is clearly noticeable by considering that the highest reducing ability of legumes is fourfold lower than the lowest value showed by insects. The digestion process did not induce any significant variation between FRAP values of digesta with respect to the aqueous extract from the same insect, excepting in silkworm, which showed a reduction of about a 25%. Conversely, digesta from all legumes showed a higher reducing ability with respect to the corresponding aqueous extract.

### 3.2. Mealworms inhibited the increase of ROS production values on colonic cells, followed by giant worms and silkworms

**Figure 2:**
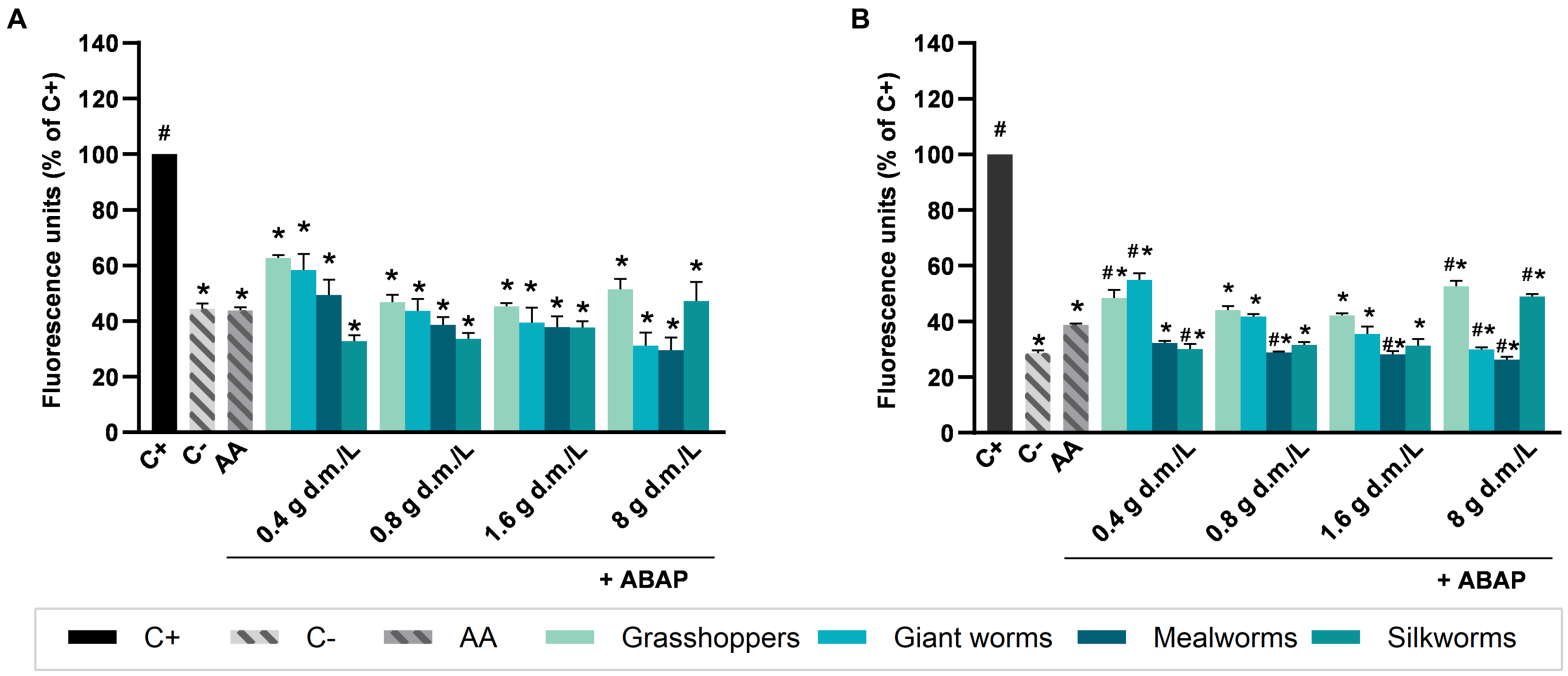
Effect of edible insects extracts on chemically induced intracellular reactive oxygen species (ROS) production in NCM460 cells. Values are expressed as percentage of fluorescence induced by ROS respect to the positive control (C+), measured after 40 min of contemporary treatment with aqueous extracts **(A)** or digesta **(B)** of edible insects and 2,2′-azobis 2-amidinopropane dihydrochloride (ABAP) 250 μM. C+: treatment with ABAP 250 μM, positive control; C−: vehicle, negative control; AA: treatment with ABAP 250 μM and ascorbic acid 2 μM. Each column represents the mean ± SEM (*n* = 3). Concentrations of extracts are expressed as g d.m./L. ^*^p < 0.05 versus C+; ^#^*p* < 0.05 versus AA, according to one-way ANOVA followed by Bonferroni’s *post hoc* analysis.

Figure 2 shows the intracellular reactive oxygen species (ROS) production values of NCM460 cells after the treatment with water extracts (Figure 2A) or digesta (Figure 2B) from edible insects in presence of 250 μM ABAP. All the aqueous extracts and digesta from insects at all the applied concentrations were able to significantly reduce ROS production induced by ABAP treatment (*p* < 0.05). Among the extracts (Figure 2A), the range of ROS production ranged from 29.46% (mealworms at 8 g d.m./L) to 62.71% (grasshoppers at 0.4 g d.m./L). Aqueous extracts from all the insects used at all the concentrations had an effect comparable to that from ascorbic acid, showing no significant differences in ROS production with respect to AA. Among the digesta (Figure 2B), the range of ROS production ranged from approximately 26% (mealworms at 8 g d.m./L) to 55% (grasshoppers at 8 g d.m./L). Mealworms at 0.8, 1.6, and 8 g d.m./L presented a significantly lower ROS production compared to AA, as well as silkworms at 0.4 g d.m./L and giant worms at 8 g d.m./L. Giant worms at 0.4 and 8 g d.m./L induced higher ROS production respect to AA, similar to giant worms at 0.4 g d.m./L and silkworms at 8 g d.m./L, *p* < 0.05.

Antioxidant activity of mealworms and giant worms, both tested as extract or digesta, improved coherently with increasing concentrations in a dose-dependent manner, while grasshoppers showed a better efficacy at intermediate concentrations (0.8 and 1.6 g d.m./L). Among the tested insects, the most effective for their antioxidant capacity was mealworm, which at 8 g d.m./L of both extract and digesta produced, respectively, a 29.24 and 26.35% of ROS with respect to the positive control. On the contrary, the lowest values of ROS were found for grasshoppers at all the concentrations. Comparing the results from each single insect, at the same concentration, we found no significant differences between aqueous extracts and digesta; the only exception was for mealworms and grasshoppers tested at 0.4 g d.m./L, which antioxidant activity was higher after digestion compared to aqueous extracts.

**Figure 3:**
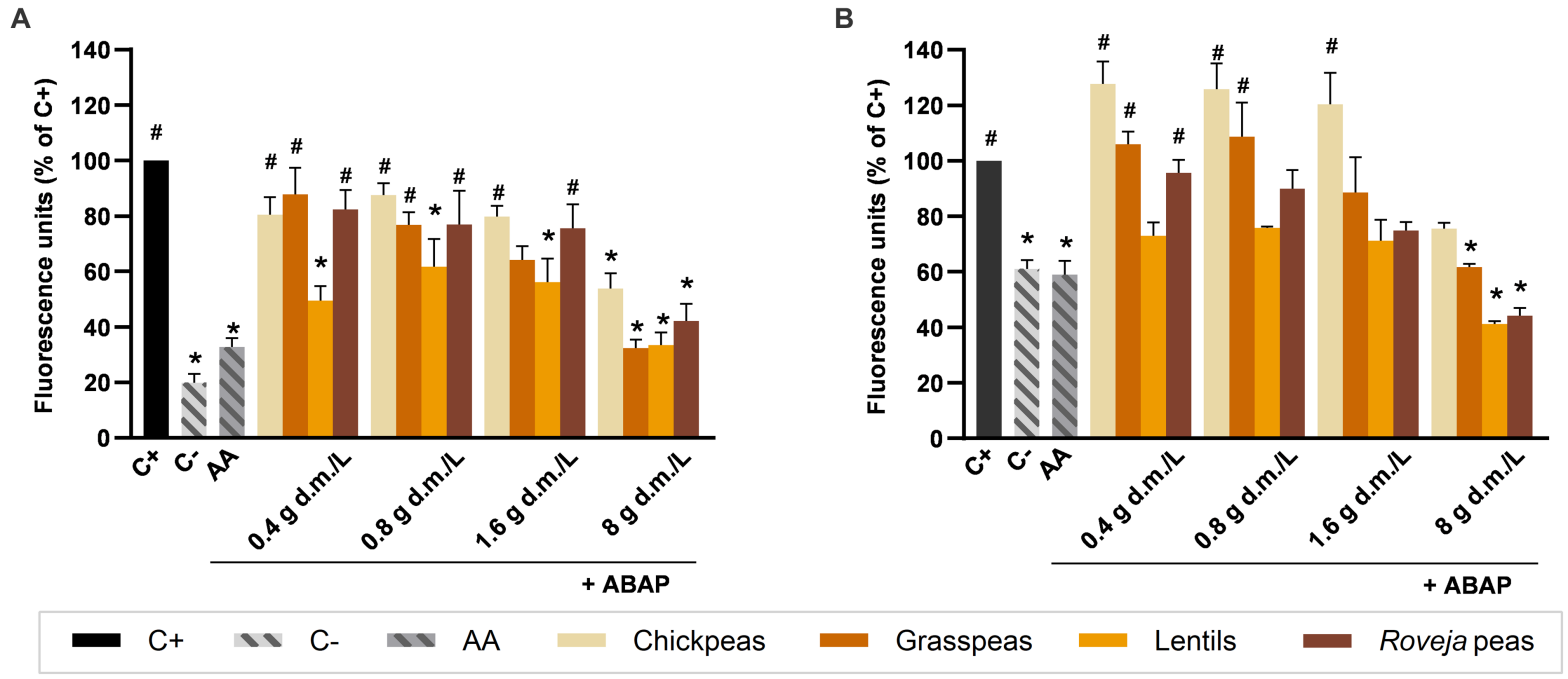
Effect of cooked legumes extracts on chemically induced intracellular reactive oxygen species (ROS) production in NCM460 cells. Values are expressed as percentage of fluorescence induced by ROS respect to the positive control (C+), measured after 40 min of contemporary treatment with aqueous extracts **(A)** or digesta **(B)** of cooked legumes and 2,2′-azobis 2-amidinopropane dihydrochloride (ABAP) 250 μM. C+: treatment with ABAP 250 μM, positive control; C−: vehicle, negative control; AA: treatment with ABAP 250 μM and ascorbic acid 2 μM. Each column represents the mean ± SEM (*n* = 3). Concentrations of extracts are expressed as g d.m./L. ^*^p < 0.05 versus C+; ^#^*p* < 0.05 versus AA, according to one-way ANOVA followed by Bonferroni’s *post hoc* analysis.

Intestinal cells treated with aqueous extracts (Figure 3A) or digesta (Figure 3B) of cooked legumes produced different ROS levels in response to induced oxidative stress. Aqueous extracts from lentils showed significantly lower levels of ROS in comparison to C+ at all the tested concentrations, leading to a range of values between about 33 and 62%, together with *Roveja* peas (42.14%), chickpeas (53.78%), and grass peas (32.37%) when used at 8 g d.m./L. By contrast, aqueous extracts from legumes did not differ for ROS production levels compared to AA at any concentration. Among the digesta, the antioxidant effect of lentils was lost, with the exception of the 8 g d.m./L that maintained a similar antioxidant activity, with ROS values of 41.18%. *Roveja* peas and grass peas at 8 g d.m./L were again effective, while chickpeas were not able to induce a significant reduction of ROS levels at any concentration. Moreover, a slight but not significant increase in ROS production respect to C+ was observed at 0.4, 0.8, and 1.6 g d.m./L, with values of 128, 126, and 120%, respectively. In both aqueous extracts and digesta, the increase of the concentration led to an improvement of the antioxidant capacity compared with the ABAP effect.

When comparing the results obtained by the same legume after aqueous extraction of digestion, few significant differences were found: chickpeas at 0.4 (*p* < 0.01), 0.8 e 1.6 g d.m./L aqueous extracts (*p* < 0.05) strongly inhibited the ROS production with respect to the digested samples.

### 3.3. Edible insects and cooked legumes reduce ROS production with different efficacy

**Figure 4:**
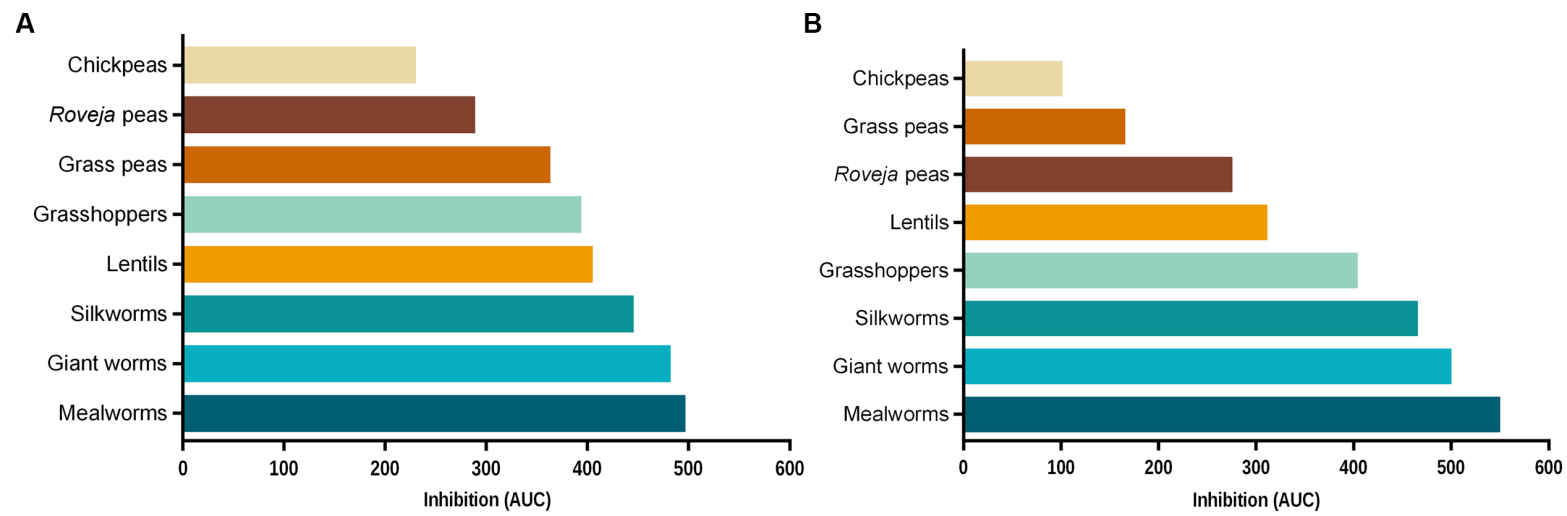
Inhibition of chemically induced intracellular reactive oxygen species (ROS) production in NCM460 cells exerted by food extracts. Percentages of fluorescence induced by ROS respect to the positive control (C+), measured after 40 min of contemporary treatment with aqueous extracts or digesta of foods and 2,2′-azobis 2-amidinopropane dihydrochloride (ABAP) 250 μM, were measured and, for each aqueous extract **(A)** or digested food **(B)**, plotted against the respective concentration used (0.4, 0.8, 1.6, and 8.0 g/L). The area under each of these curves, defined as the region bounded by each curve and the *x*-axis, was calculated and reported. Each bar represents the average of three independent experiments. C+: treatment with ABAP 250 μM, positive control.

In order to compare the efficacy of the extracts among them, Figure 4 reports the reduction of ROS levels released by NCM460 cells following the treatment with aqueous extracts (Figure 4A) and digesta (Figure 4B) of edible insects and legumes, expressed as area under the curve. Results highlighted that edible insects and cooked legumes were able to reduce ROS production respect to the ABAP treatment, with a different efficacy.

Regarding the digested samples, edible insect showed a higher inhibition ability with respect to legumes, while for the aqueous extracts the trend was similar for both groups, except for cooked lentils, which have an inhibition activity comparable to insects. Chickpeas were the less performing both digesta and aqueous extracts, while the best results were detected for mealworms. Moreover, antioxidant activity induced from digested chickpeas and grass peas was remarkably lower than the respective aqueous extracts.

## 4. Discussion

The main findings of the present study suggest that extracts of edible insects, novel source of animal proteins and bioactive compounds, exert interesting antioxidant properties *in vitro* on a classical intestinal cellular model, comparable or even higher than well-known vegetable sources of bio-actives ingredients, such as legumes. A previous review study pointed the existing evidences in literature regarding the antioxidant activity exerted by different insects, mostly *in vitro* (14). However, in order to introduce them as part of a dietary pattern in a near future, it remains necessary a comparison with other foods with similar and comparable nutritional and ecological impacts. In this context, legumes stand as the best option to evaluate the antioxidant activity of edible insects, since legumes are considered as traditional alternative protein sources with respect to the conventional livestock products and as a source of bioactive compounds with potential antioxidant activity (17, 18). The *in vitro* antioxidant activity of aqueous extracts from edible insects, measured using the FRAP method, showed a remarkable higher reducing activity than legumes. In a previous study from our laboratory (19), FRAP analysis of several insects and invertebrates showed values of about 2 mmol Fe^2+^/100 g d.m. for aqueous extracts of grasshoppers and 1 mmol Fe^2+^/100 g d.m. for silkworm and mealworms. The FRAP values recorded in the present study for mealworms and grasshoppers are also comparable to results reported in Di Mattia et al. (19), while silkworms showed here a high difference, probably due to the variability on the different rearing conditions or drying treatment of the product by the different sellers, which could have an impact on the bioactive amounts and activity.

In the present study, an *in vitro* digestion procedure was used to assess whether the oral, gastric, and intestinal phases of digestion affected the antioxidant activity of the samples, which is mandatory to evaluate the biological activity of foods and their components. When comparing aqueous extracts and digesta from the tested insect samples, no significant differences were found in the antioxidant capacity and protection from ROS production, except for silkworms digesta, which exerted a lower activity than that from the aqueous extract. Conversely, digestion process enhanced the reducing activity of all the legumes. Regarding the pulses, Gallego et al. (24) reported a significant increase in FRAP values after *in vivo* digestion of cooked pastes of lentils and peas. To the best of our knowledge, data on the effect of *in vitro* digestion using INFOGEST method on the antioxidant capacity of edible insects with respect to non-digested insects are not available to date; most of the information regarding this topic is focused on the hydrolysates from protein fractions, leading to an increased antioxidant activity (25, 26). However, in this study, whole edible insects were digested using a different pattern of enzymes, thus the higher complexity of the matrix and the method could had led to these results.

To confirm the *in vitro* reducing activity in a biological model, aqueous extracts and digesta from legumes and edible insects were tested for their ability to mitigate and prevent ROS production in the NCM460 cell line. While edible insects were able to counteract the increasing of ROS production induced by ABAP, without showing the high difference observed in FRAP levels, legumes were clearly less effective. The slight increase of antioxidant activity of legumes observed using the FRAP method was not confirmed in the cellular model; indeed, digestion process induced a loss of the antioxidant capacity in lentils. Due to the need of carrying out the digestive process, the higher stability showed by edible insects respect to legumes represents a clear advantage.

Overall, edible insects showed a higher reducing power and were more effective in modulating the redox status in cellular model of oxidative stress in comparison with a conventional alternative source of proteins such as legumes. Of note, these properties were maintained after digestion. Both foods were tested in realistic conditions: legumes were soaked and cooked using replicable methods in a domestic context, while dried edible insects did not require further processing to be eaten.

Antioxidant-rich foods play an important role in the prevention of oxidative stress-related diseases. Specifically, intestinal oxidative stress contributes to the development of pathologies, such as gluten-related diseases, inflammatory bowel disease, and colorectal cancer (27), as well as an intestinal dysbiosis (28). The promising preliminary results obtained in this study suggest a potential role for edible insects consumption in the prevention of oxidative stress-related intestinal diseases, and such results deserve to be confirmed through well-designed clinical studies. Intriguingly, edible insects as silkworms (29) can contain bioactive compounds as 1-Deoxynojirimycin, an alkaloid commonly found in mulberry leaves. This compound exerts its activity by lowering the blood glucose (30), and it is also necessary for the improvement of the cellular antioxidant status (31). This aspect suggests that, since antioxidant-rich foods or beverages consumed during mealtime can help in counteracting the non-physiological alteration of the endogenous redox homeostasis (32), edible insects could help to restore redox balance in a similar manner.

Although entomophagy is not a common practice in Western countries at present, its growing interest due to the aforementioned advantaged prompted an update of the European Food Legislation for the edible insects consumption. The European Food Safety Authority (EFSA) issued a Scientific Opinion on a risk profile related to production and consumption of insects as food and feed, concluding that biological and chemical hazards of edible insects could be influenced mainly by the specific production and processing methods, the substrate used, the stage of harvest, the insect species, and highly recommending the production of data on these specific issues (33). It states also that when currently allowed feed materials are used as substrate to feed insects, the possible occurrence of microbiological hazards is likely to be equivalent to their occurrence in other non-processed sources of protein of animal origin (33). Currently, the production and marketing of insects as food in Europe is governed by Regulation (EU) No 2015/2283 (34) that applies to novel foods, i.e., foods that were not significantly used for human consumption within the European Union before 1997. To date, only three species received the authorization to be sold on the market for human consumption within the European Union: *Tenebrio molitor* (mealworms) (35), *Locusta migratoria* (grasshoppers) (36), and *Acheta domesticus* (crickets) (37), all of them approved in the last 2 years. Disgust is still a common reaction induced by the idea of eating insects in westerners: among the different solutions to further increase acceptability of edible insects as food, adding further information on health benefits of edible insects through scientific studies and further dissemination of the result could exert a positive effect in the population (38).

This study stands out the antioxidant capacity of edible insects, which was found potentially higher compared to traditional vegetal protein sources as legumes. Such results strengths the concept that the inclusion of edible insects in daily dietary patterns may serve as: (i) an alternative intake of animal-based proteins with low ecological impact; and (ii) an increased intake of bioactive compounds, which have a potential antioxidant capacity on human body. However, in order to fully elucidate and endorse the antioxidant properties of edible insects, further steps are needed. Among these, food industries should focus the attention in conceiving and producing insects-containing foods able to preserve the antioxidant capacity showed in *in vitro* assays. Then, well designed and human intervention studies including the consumption of insects-containing foods are needed to translate the research from bench assays, offering an integrated explanation for the modulation of the *in vivo* oxidative stress and presenting potential anti-inflammatory markers.

## Data availability statement

The raw data supporting the conclusions of this article will be made available by the authors, without undue reservation.

## Author contributions

MS: conceptualization and supervision. VD’A and NB: methodology. VD’A: formal analysis and writing—original draft preparation. VD’A, DA, and MS: investigation. VD’A, NB, CM, GS, MR-S, RP, DA, and MS: writing—review and editing. All authors contributed to the article and approved the submitted version.

## Funding

This research was funded the European Union—Next Generation EU. Project Code: ECS00000041; Project CUP: C43C22000380007; Project Title: Innovation, digitalization, and sustainability for the diffused economy in Central Italy—VITALITY.

## Conflict of interest

The authors declare that the research was conducted in the absence of any commercial or financial relationships that could be construed as a potential conflict of interest.

## Publisher’s note

All claims expressed in this article are solely those of the authors and do not necessarily represent those of their affiliated organizations, or those of the publisher, the editors and the reviewers. Any product that may be evaluated in this article, or claim that may be made by its manufacturer, is not guaranteed or endorsed by the publisher.

## Abbreviations

AA: Treatment with ABAP 250 μM and ascorbic acid 2 μM
ABAP: 2,2′-Azobis 2-amidinopropane dihydrochloride
ANOVA: Analysis of variance
AUC: Area under the curve
C−: vehicle (negative control)
C+: Treatment with ABAP 250 μM (positive control)
DCF-DA: Dichlorofluorescein diacetate
EFSA: European Food Safety Authority
FRAP: Ferric reducing antioxidant power
GHG: Greenhouse gas
HBSS: Hanks’ balance salt solution
ROS: Reactive oxygen species
