# Supplementary material for: Edible insects and legumes exert an antioxidant effect on human colon mucosal cells stressed with 2,2′-azobis (2-amidinopropane)-dihydrochloride
Source: Front Nutr. 2023 Jul 6;10:1219837. doi: 10.3389/fnut.2023.1219837 (PMC10358759; doi:10.3389/fnut.2023.1219837)

Supplementary Figure 1. Graphical representation of the considered insect and legume samples, the extraction and digestion procedures and the relative cell treatments.

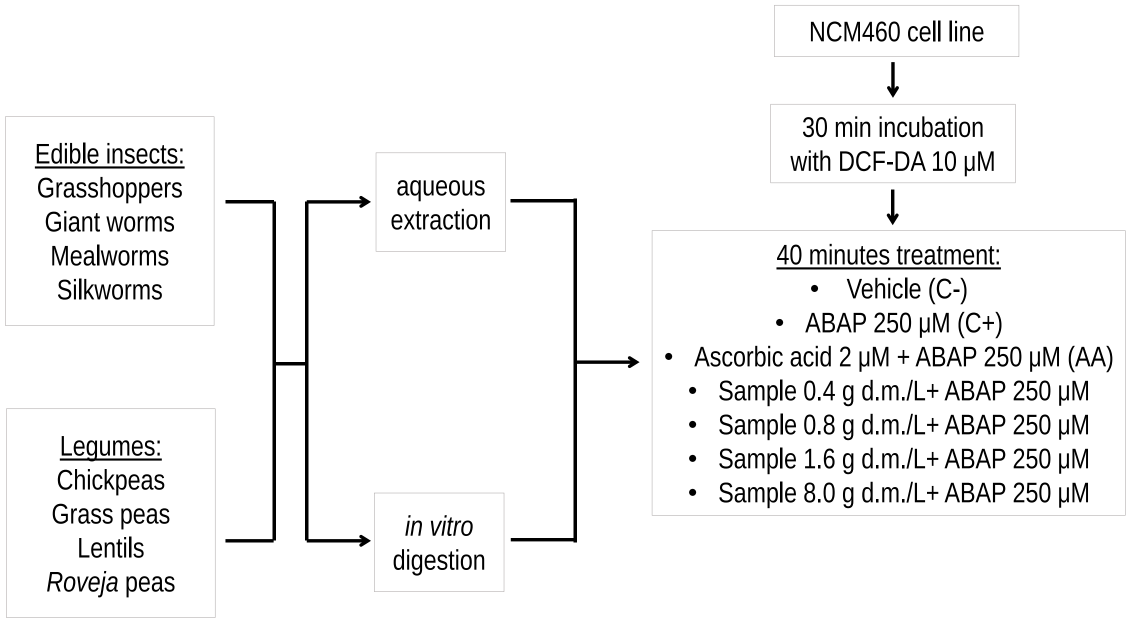

Supplement: Supplementary file 1 [file Data_Sheet_1.PDF]
